# Cat or Dog Ownership and Seroprevalence of Ehrlichiosis, Q Fever, and Cat-Scratch Disease

**DOI:** 10.3201/eid0910.030206

**Published:** 2003-10

**Authors:** Martina Skerget, Christoph Wenisch, Florian Daxboeck, Robert Krause, Renate Haberl, Doris Stuenzner

**Affiliations:** * University Hospital Graz, Graz, Austria; † University Hospital, Vienna, Austria

## Abstract

Concerns have been raised about the role of domestic cats or dogs in the acquisition of zoonoses, in particular in pregnant women or immune-suppressed persons. We report that cat or dog ownership is not associated with an increased seroprevalence of antibodies to *Anaplasma phagozytophilum*, *Coxiella burnetii*, and *Bartonella henselae* in symptom-free persons in Styria, Austria.

Keeping pet cats and dogs is very popular in Austria. However, these animals can serve as reservoirs for the agents of important bacterial infectious diseases and as a potential source of infection for humans, even though the infectious animals may be asymptomatic. Infections are potentially transmitted from domestic animals to humans by scratches, bites, or close contact. Examples for such infections include human granulocytic ehrlichiosis (*Anaplasma phagocytophilum*), cat-scratch disease (CSD, *Bartonella henselae*), and Q fever (*Coxiella burnetii*).

Cats are known to be the most important source of infections for B. henselae (aeroprevalence in Austrian cats is 33%) (*1*). However, dogs may also transmit *B. henselae* (*2*). Animals are contagious through their blood, which may contaminate saliva in cases of gum bleeding. Fleas from infected animals may contain the infectious agent, and bites from these fleas can transmit CSD. Typically, CSD is a benign and self-limiting disease in humans, occurring with lymphadenopathy, low-grade fever, primary cutaneous inoculation lesion, and weight loss, lasting 6–12 weeks. Rarely observed atypical signs and symptoms include erythema nodosum, figurate erythemas, thrombocytopenic purpura, Perinaud’s oculoglandular syndrome, encephalopathy, hepatic granulomas, osteomyelitis, pulmonary disease, and optic neuritis (*3*). These severe manifestations occur in immunocompetent patients, whereas bacillary angiomatosis or peliosis hepatitis are more likely to develop in immunosuppressed patients.

*C. burnetii* infection has been associated with a chronic fatigue–like syndrome (*4*). Both cats and dogs are well-described reservoirs for *C. burnetii* (*5*). In humans, *C. burnetii* infection usually is asymptomatic (60%) or manifests as a mild disease with fever, headache, myalgias, and spontaneous recovery (*5*). However, this infection may lead to serious complications and even death in patients with acute disease, especially those with meningoencephalitis and myocarditis and, more frequently, in chronically infected patients with endocarditis. Q fever in pregnancy has been associated with abortion, premature birth, and low weight in newborns (*6*,*7*).

Within the past several decades, the number of *Ehrlichia* and *Anaplasma* spp. recognized to infect cats, dogs, and humans has expanded substantially (*8*). The agent of human granulocytic ehrlichiosis (HGE) has recently been classified as *A. phagozytophilum* (*9*). The disease has influenzalike symptoms with variable degrees of anemia, thrombocytopenia, leukopenia, and elevated liver enzymes. Dogs are thought to be sentinels for assessing risk for HGE in humans (*10*). Cases of HGE in the United States are increasing in incidence (*11*). Reports of acute cases in Europe have been rare, although serosurveys of the prevalence of antibodies to *A. phagocytophilum* have been conducted. A survey in Slovenia showed that 15.4% of the examined population had detectable antibodies to the pathogen and several cases of HGE had been confirmed (*12*). No similar serosurvey has been conducted in Austria, although *Ixodes ricinus*, thought to be the principal vector in Europe, is present in Austria (*13*). We report that the rate of seropositivity of *A. phagocytophilum* (immunoglobulin [Ig] G antibodies 25/376 [7%], IgM antibodies 6/376 [2%]), *B. henselae* (88/376 [23%]), and *C. burnetii* (23/376 [6%]) in Styria, Austria, is not affected by cat or dog ownership.

## The Study

We examined serum specimens from 376 persons that were collected at the University Hospital Graz, Austria, from December 2001 to April 2002. These persons had no history of a tick bite for at least 1 year. A total of 202 persons with domestic dogs, cats, or both (dogs n=77, cats n=106, dogs and cats n=19) were compared with 174 persons who had no domestic pet contact for at least 1 year. Study participants in the no-pet group had never lived with a cat or a dog. The domestic pets had no symptoms or signs of infection, as determined by veterinarians. All participants were outpatients, and blood samples were drawn for routine blood tests. Each participant completed a questionnaire about medical history. These persons had no known history of rickettsiosis, borreliosis, or tick-borne encephalitis and reported no febrile or influenzalike illness during the previous 6 months. Each participant provided verbal consent for the serum to be used for detecting antibodies against several infectious agents related to zoonoses. The following information was collected for each participant: age, sex, area of residence, and medical history. Demographic data for the participants are shown in Table 1.

**Table 1:** Demographic data of patients with and without domestic pets

	No pet (n=174)	Cat (n=106)	Dog (n=77)	Cat and dog (n=19)
Age (y)	55±19	51±14	53±20	55±15
Sex (male/female)	55/119	32/74	37/38	6/13
Urban/rural	133/41	63/43	39/36	7/12

Serum samples were tested for the infectious agents with the following methods: *B. henselae* by indirect immunofluorescence (Biognost, Gräfelfing, Germany), *C. burnetii* by microagglutination reaction (Bodybion Mar; Bioveta National Enterprise Nitra, Slovakia), and HGE IgM indirect immunofluorescent antibody test (IFA, MRL Diagnostics, Cypress, CA), HGE by IFA IgG (Focus Technologies, Cypress, CA) with titers >64 were considered positive. All assays were performed in duplicate according to manufacturers’ instructions. Biostatistical analysis was performed with the statistical package Jandel SigmaStat Statistical Software, version 2.0, NL (Jandel SigmaStat, Jandel, San Rafael, CA). Chi-square tests or t tests were used to determine differences between the presence of antibodies to the test organisms and demographic data, as appropriate. A two-tailed p value <0.05 was considered significant.

A total of 88 (23%) of 376 persons had antibodies against *B. henselae*. No differences in terms of age, sex, urban or rural residence, or concomitant diseases were noted. No difference in persons with and without domestic pets (46/174 [26%] and 42/202 [20%], respectively) was seen (Table 2).

**Table 2:** Seroprevalence of antibodies against *Anaplasma phagocytophilum, Coxiella burnetti*, and *Bartonella henselae* in persons with and without domestic pets^a^

Organism	No pet n=174 (%)	Cat n=106 (%)	Dog n=77 (%)	Cat and dog n=19 (%)	No pet/cat p value	No pet/dog p value	No pet/cat and dog p value
*A. phagocytophilum*							
IgG^b^	12 (6)	7 (7)	2 (5)	4 (13)	0.239	0.332	0.162
IgM	2 (1)	1 (1)	1 (2)	2 (6)	0.856	0.849	0.611
*C. burnetii*	13 (6)	6 (6)	2 (5)	2 (6)	0.235	0.293	0.257
*B. henselae*	46 (26)	29 (28)	9 (23)	4 (13)	0.272	0.143	0.113

^a^No differences are evident by chi-square test for comparisons between the groups for all pathogens. ^b^Ig, immunoglobulin.

The overall prevalence of antibodies to *C. burnetii* was 6% (23/376 persons). Again, no differences in age, sex, urban or rural residence, or concomitant diseases (13/202 [6%]) and persons with and without domestic pets (10/174 [6%]), were seen (Table 2).

The overall prevalence of IgG/IgM antibodies to *A. phagocytophilum* was 7% and 2% (25/376, and 6/376, respectively). No differences in terms of age, sex, urban or rural residence, or concomitant diseases were seen. IgG antibody titers were low (of 25 positive serum specimens, 17 had titers >64, and 8 had titers >128). In the six patients with positive IgM antibodies, no clinical evidence of ehrlichiosis was present, and additional blood samples showed no cytopenia. No difference in persons with (8% and 2% IgG/IgM positive, respectively) and without domestic pets (6% and 1% IgG/IgM positive, respectively) was seen (Table 2).

The seroreactivity to *C. burnetii* and *B. henselae* did not differ between *A. phagocytophilum–*positive patients and *A. phagocytophilum–*negative patients (p>0.05). Likewise, the seroreactivity to *A. phagocytophilum* and *B. henselae* did not differ between *C. burnetii*–positive patients and *C. burnetii*–negative patients (p>0.05). In addition, the seroreactivity to *A. phagocytophilum* and *C. burnetii* did not differ between *B. henselae–*positive patients and *B. henselae*–negative patients (p>0.05).

## Conclusions

Veterinarians have the responsibility of providing accurate information to their clients about the zoonotic transmission of infections from pets, especially to those most vulnerable, such as children, pregnant women, the elderly, and the immunocompromised. Effective education is vital to allay public concerns and promote responsible pet ownership (*14*). With respect to *A. phagocytophilum*, *B. henselae*, and *C. burnetii,* cat or dog ownership was not related to an increased incidence of antibodies in our study.

CSD has been reported worldwide and seems to be the most common *B. henselae* infection in humans. In the United States, epidemiologic databases estimate that approximately 24,000 cases of CSD occur each year, with a calculated incidence of 9.3/10,000 ambulatory patients per year. In various studies, the seroprevalence of antibodies to *B. henselae* in humans ranges from 3.6% to 6% (*15*–*17*). Although CSD may occur in persons of any age, most patients are <18 years of age, perhaps because children are more likely to have close and rough contact with cats. The observed high incidence of antibody positivity in adults could be related to the persistence of antibodies after asymptomatic infection. Alternatively, it may be due chronic low-grade infection, which has been demonstrated for *B. quintana* and *B. bacilliformis* (*18*,*19*). The incidence of CSD is seasonal; most cases occur in August to October in northern temperate areas. The prevalence of the disease also varies by geographic location. The prevalence of antibodies to *B. henselae* is reportedly higher in areas with warm humid climates, where the prevalence and intensity of cat flea infestations are higher (*20*). Cats may infect humans either directly through scratches and bites or indirectly by means of the cat flea (*Ctenocephalides felis*), which is the arthropod vector (*20*). Recent research has demonstrated that *B. henselae* seroprevalence is elevated in patients with coronary vascular disease (*21*). Considering the comparatively high mean age of the patients tested in this study and the high percentage of patients admitted for cardiovascular disease (Table 1), our results provide indirect support for this finding.

Because Q fever is rarely a notifiable disease, its incidence in humans cannot be assessed in most countries. Current epidemiologic studies indicate, however, that Q fever should be considered a public health problem in many countries, including France, United Kingdom, Italy, Spain, Germany, Israel, Greece, and Canada (Nova Scotia), as well as in many countries where Q fever is prevalent but unrecognized because of poor surveillance. Q fever remains primarily an occupational hazard in persons in contact with domestic animals such as cattle, sheep, and, less frequently, goats. Persons at risk from Q fever include farmers, veterinarians, abattoir workers, those in contact with dairy products, and laboratory personnel performing *C. burnetii* culture and working with *C. burnetii*–infected animals. However, reports of sporadic cases in persons living in urban areas after occasional contact with farm animals or after contact with infected pets such as dogs and cats have increased. Our data suggest that Q fever also occurs in Austria but that pet ownership or rural residence has no effect on seroprevalence. *C. burnetii* has also been isolated from the locally widespread *I. ricinus* ticks (*22*), although no tick-borne Q fever has been described in Austria.

For *A. phagocytophilum,* our seroprevalence is similar to those observed in Greece, much lower than that in Slovenia (15.4%), and higher than those seen in Bulgaria (2.9%) and Germany (1.9%) (*23*). In Austria (Styria), the prevalence in blood donors is 4% (*24*). We agree with Daniel et al. (*23*) that this Austrian prevalence could be due to the fact that the prevalence in blood donors is lower, unlike the survey in Greece, Slovenia, or this assessment. Even though the titers to *A. phagocytophilum* were low in our study, they suggest infection at an undetermined time.

Our data also suggest a discrepancy between the comparatively high seroprevalence of specific antibodies to *A. phagozytophilum*, *C. burnetii*, and *B. henselae*, respectively, and the few diagnosed cases of human disease. Clearly, cat or dog ownership is not related to an increased incidence of antibodies against these pathogens. However, because of the widespread distribution of *I. ricinus*, an organism also capable of transmission, this finding must be interpreted cautiously. In addition, the small sample size may have been insufficient to detect differences between the groups. Hence, recommendations for the population at risk for a severe course of these infections (pregnant women, the elderly, immunosuppressed patients, HIV-positive persons, and infants) are not possible. Nonetheless, such groups of patients may benefit from efficient tick and flea control on dogs and cats, as an adjunct to decrease mechanical transport of the parasites into their homes. Further research is needed to clarify the importance of pets or ticks for these diseases in Austria.
